# Involvement of Receptor for Advanced Glycation Endproducts in Hypertensive Disorders of Pregnancy

**DOI:** 10.3390/ijms20215462

**Published:** 2019-11-01

**Authors:** Juria Akasaka, Katsuhiko Naruse, Toshiyuki Sado, Tomoko Uchiyama, Mai Makino, Akiyo Yamauchi, Hiroyo Ota, Sumiyo Sakuramoto-Tsuchida, Asako Itaya-Hironaka, Shin Takasawa, Hiroshi Kobayashi

**Affiliations:** 1 Department of Obstetrics and Gynecology, Nara Medical University, 840 Shijo-cho, Kashihara, Nara 634-8522, Japan; juria@naramed-u.ac.jp (J.A.); naruse@naramed-u.ac.jp (K.N.); tsado@naramed-u.ac.jp (T.S.); hirokoba@nmu-gw.naramed-u.ac.jp (H.K.); 2 Department of Biochemistry, Nara Medical University, 840 Shijo-cho, Kashihara, Nara 634-8521, Japan; uchiyama0403@naramed-u.ac.jp (T.U.); m.makino@naramed-u.ac.jp (M.M.); yamauchi@naramed-u.ac.jp (A.Y.); hiroyon@naramed-u.ac.jp (H.O.); ssumiyo@naramed-u.ac.jp (S.S.-T.); iasako@naramed-u.ac.jp (A.I.-H.)

**Keywords:** hypertensive disorders of pregnancy, RAGE, AGE, adipocyte, IL-6, CCL2, LPS

## Abstract

Preeclampsia/hypertensive disorders of pregnancy (PE/HDP) is a serious and potentially life-threatening disease. Recently, PE/HDP has been considered to cause adipose tissue inflammation, but the detailed mechanism remains unknown. We exposed human primary cultured adipocytes with serum from PE/HDP and healthy controls for 24 h, and analyzed mRNA expression of several adipokines, cytokines, and ligands of the receptor for advanced glycation endproducts (RAGE). We found that the mRNA levels of interleukin-6 (*IL-6*), C-C motif chemokine ligand 2 (*CCL2*), high mobility group box 1 (*HMGB1*), and *RAGE* were significantly increased by the addition of PE/HDP serum. Among RAGE ligands, advanced glycation endproducts (AGE) and HMGB1 increased mRNA levels of *IL-6* and *CCL2* in SW872 human adipocytes and mouse 3T3-L1 cells. The introduction of small interfering RNA for *RAGE* (siRAGE) into SW872 cells abolished the AGE- and HMGB1-induced up-regulation of IL-6 and CCL2. In addition, lipopolysaccharide (LPS), a ligand of RAGE, increased the expression of IL-6 and CCL2 and siRAGE attenuated the LPS-induced expression of IL-6 and CCL2. These results strongly suggest that the elevated AGE, HMGB1, and LPS in pregnant women up-regulate the expression of IL-6 and CCL2 via the RAGE system, leading to systemic inflammation such as PE/HDP.

## 1. Introduction

Preeclampsia/hypertensive disorders of pregnancy (PE/HDP) is a serious and potentially life- threatening disease appearing as a complication in about 2–12% of all pregnancies and associated with significant perinatal and maternal mortality [1,2]. It is estimated that more than 60,000 women worldwide die of the disease each year; it is one of the main causes of maternal mortality [3]. There is considerable evidence that maternal obesity, increased insulin resistance, inflammation, and aberrant fatty acid metabolism are involved in the pathogenesis of PE/HDP [4,5]. Inflammatory reactions have recently been attracting attention as the pathophysiological characteristics of PE/HDP, including vascular endothelial dysfunction and placental abnormalities [6,7,8,9,10,11,12,13,14]. Shallow trophoblast invasion and inadequate artery remodeling in pregnancy may cause placental hypoperfusion, hypoxia, or ischemia, which play an important role in the pathogenesis of PE/HDP [15]. The link between adiposity, inflammation, and insulin resistance has been increasingly acknowledged since Spiegelman and his colleagues demonstrated the relationship [16]. White adipose tissue secretes pro-inflammatory cytokines which contribute significantly to the chronic inflammatory state and metabolic complications of obesity [17]. It is plausible that similar disturbances in adipocyte function might contribute to the development of the clinical syndrome of PE/HDP, a state of inflammation and insulin resistance.

Adipose tissue, complex tissue composed of preadipocytes, adipocytes, and stromal vascular cells, is one of the representative organs to contribute to worsening insulin resistance through inflammation and subsequent dysfunction. Visceral adiposity correlates with metabolic risk factor [18] and adverse metabolic outcomes in pregnancy including gestational diabetes mellitus and PE/HDP [19,20,21]. Adipokines are cytokines expressed in and secreted from adipocytes in response to the systemic nutritional status, and some of them induce macrophage infiltration and inflammatory cytokine secretion [22,23]. In the present study, we analyzed expression of adipokines including inflammatory cytokines in adipocytes and found the involvement of receptor for advanced glycation endproducts (RAGE) in expression of interleukin-6 (IL-6) and C-C motif chemokine ligand 2 (CCL2) in adipocytes.

## 2. Results

### 2.1. PE/HDP Patient Sera Up-Regulated Gene Expression of IL-6, CCL2, High Mobility Group Box (HMGB)1, S100 Ca^2+^-Binding Protein B (S100B), and Receptor for Advanced Glycation Endproducts (RAGE) in Primary Cultured Human Adipocytes

**Figure 1:**
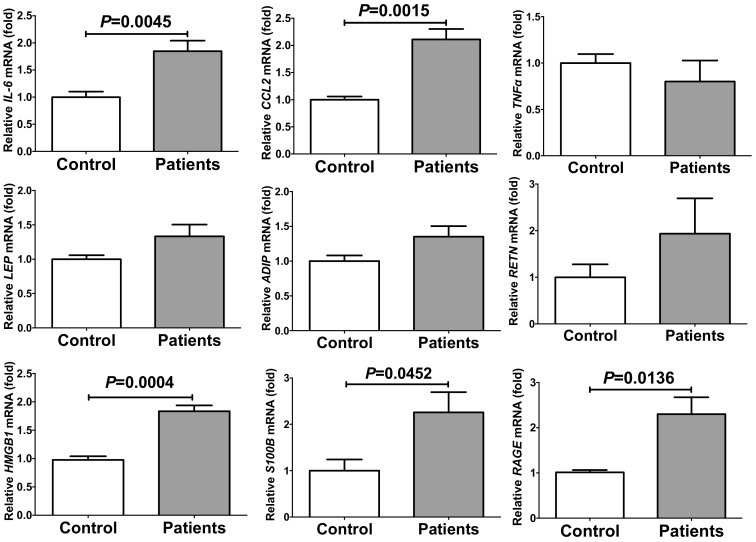
The mRNA levels of *IL-6*, *CCL2*, *TNFα*, *LEP*, *ADIP*, *RETN*, *HMGB1*, *S100B*, and *RAGE* in primary cultured human adipocytes treated with sera from disease-free control (Control) or preeclampsia/hypertensive disorders of pregnancy (PE/HDP) patients (Patients) for 24 h. The levels of the mRNAs were measured via real-time reverse transcriptase-polymerase chain reaction (RT-PCR) using *β-actin* as an endogenous control. Data are expressed as mean ± SE for each group (*n* = 4). The statistical analyses were performed using Student’s *t*-test.

Obesity increases PE/HDP risk. Maternal obesity, increased insulin resistance, and inflammation are involved in the pathogenesis of PE/HDP [24,25]. Furthermore, PE/HDP risk has been reported to increase 2–4-fold among women with diabetes [26]. We therefore hypothesized that the PE/HDP patient sera contain some of these factors that induce insulin resistance and/or inflammation. We incubated primary cultured human adipocytes with sera from disease-free pregnant women (control) or those from PE/HDP (patients) for 24 h, and the gene expression of *IL-6*, *CCL2*, *tumor necrosis factor α* (*TNFα*), *leptin* (*LEP*), *adiponectin* (*ADIP*), *resistin* (*RETN*), *HMGB1*, *S100B*, and *RAGE* in the adipocytes was measured via real-time reverse transcriptase-polymerase chain reaction (RT-PCR). As shown in Figure 1, mRNA levels of *IL-6*, *CCL2*, *HMGB1*, *S100B*, and *RAGE*, but not *TNFα*, *LEP*, *ADIP*, and *RETN* (*P* = 0.4496, *P* = 0.1157, *P* = 0.0875, and *P* = 0.2912, respectively) were significantly increased by the addition of PE/HDP patient sera compared to those cells incubated with control sera.

### 2.2. Up-Regulation of IL-6 and CCL2 by HMGB1 and Advanced Glycation Endproducts (AGE) in Adipocytes

**Figure 2:**
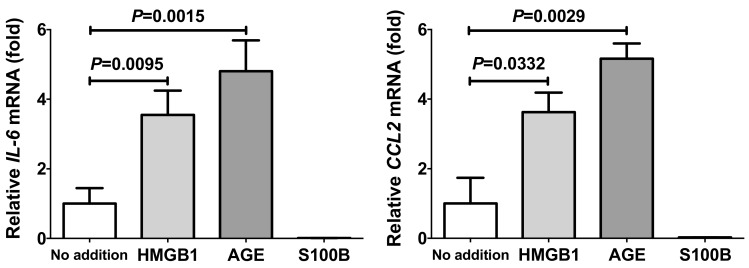
The mRNA levels of *IL-6* and *CCL2* in SW872 human adipocytes treated with 1 µg/mL HMGB1, 150 µg/mL advanced glycation endproducts (AGE), or 100 ng/mL S100B for 24 h. The levels of the mRNAs were measured via real-time RT-PCR using *β-actin* as an endogenous control. Data are expressed as mean ± SE for each group (*n* = 4). The statistical analyses were performed using Student’s *t*-test vs. No addition.

It is well-known that HMGB1 and S100B are typical ligands for RAGE. RAGE expression was reported in adipocytes and SW872 cells [27,28], and furthermore immunofluorescent staining of RAGE in 3T3-L1 adipocytes was shown [27]. RAGE expression was up-regulated by ligands for RAGE [29], we tested whether ligands for RAGE up-regulate gene expression of inflammatory mediators, such as *IL-6* and *CCL2*, in human SW872 adipocytes. We added HMGB1, AGE, and S100B in SW872 culture medium, incubated for 24 h, and the expression of *IL-6* and *CCL2* was analyzed via real-time RT-PCR. As shown in Figure 2, mRNAs of *IL-6* and *CCL2* were significantly up-regulated by the addition of HMGB1 and AGE. In contrast, S100B, another noted ligand for RAGE, failed to up-regulate mRNA for *IL-6* or *CCL2*.

**Figure 3:**
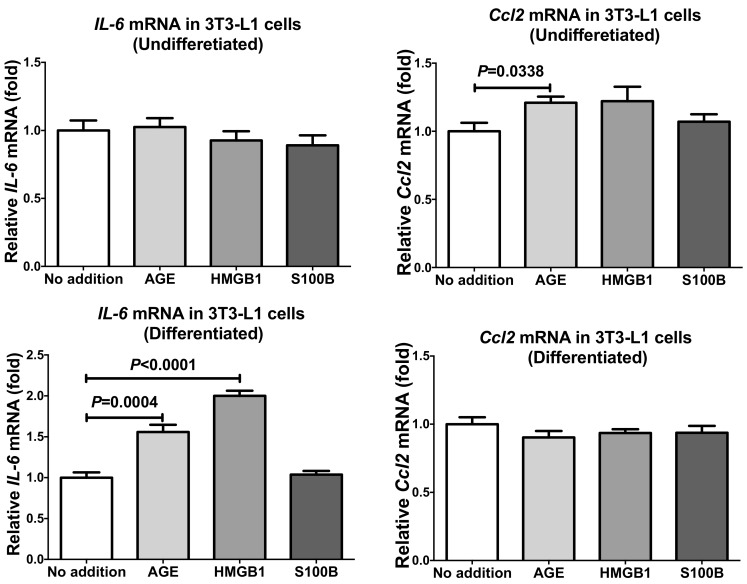
The mRNA levels of *IL-6* and *Ccl2* in 3T3-L1 mouse cells (undifferentiated preadipocytes and differentiated adipocytes) treated with 300 µg/mL AGE, 1 µg/mL HMGB1, or 100 ng/mL S100B for 24 h. The levels of the mRNAs were measured via real-time RT-PCR using *rat insulinoma gene* (*Rig*)/*ribosomal protein S15* (*RpS15*) as an endogenous control. Data are expressed as mean ± SE for each group (*n* = 4). The statistical analyses were performed using Student’s *t*-test vs. No addition.

In order to see whether the up-regulation of mRNAs for *IL-6* and *Ccl2* occurred only in SW872 or other adipocytes, we cultured mouse 3T3-L1 preadipocytes, differentiated them into differentiated adipocytes, and tested whether ligands for RAGE up-regulate gene expression of *IL-6* and *Ccl2* in mouse 3T3-L1 undifferentiated preadipocytes and differentiated adipocytes. As shown in Figure 3, the mRNA levels of *IL-6* were significantly up-regulated by AGE and HMGB1 but not by S100B (*P* = 0.6414) in differentiated 3T3-L1 adipocytes, but unchanged by any of the RAGE ligands (AGE, HMGB1, or S100B) in undifferentiated preadipocytes (*P* = 0.8037 [No addition vs. AGE], *P* = 0.4793 [No addition vs. HMGB1], and *P* = 0.3138 [No addition vs. S100B]). In contrast, the mRNA levels of *Ccl2* remained unchanged in response to AGE, HMGB1, or S100B in 3T3-L1 differentiated adipocytes (*P* = 0.1892 [No addition vs. AGE], *P* = 0.2885 [No addition vs. HMGB1], and *P* = 0.4024 [No addition vs. S100B]), but significantly up-regulated in the undifferentiated preadipocytes by the addition of AGE but not by HMGB1 and S100B (*P* = 0.1241 [No addition vs. HMGB1] and *P* = 0.4305 [No addition vs. S100B]) (Figure 3). Previous studies reported that S100B up-regulated TNFα in adipocytes [30,31]. In contrast, S100B induced neither *IL-6* nor *CCL2* in adipocytes in this study, suggesting that SW872 and 3T3-L1 cells may insensitive to S100B.

### 2.3. Down-Regulation of RAGE Attenuated the Increases of IL-6 and CCL2 in Adipocytes Treated with Small Interfering RNA (siRNA) for RAGE

**Figure 4:**
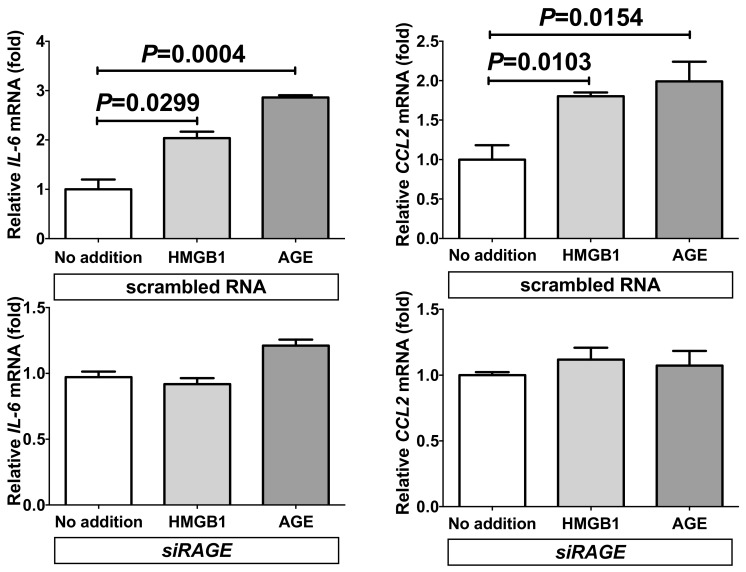
Effects of siRNA against *RAGE* on HMGB1- and AGE-induced gene expression of *IL-6* and *CCL2*. SiRNA for *RAGE* was transfected into SW872 cells and the cells were incubated with HMGB1 or AGE for 24 h. The levels of *IL-6* and *CCL2* mRNA were measured via real-time RT-PCR using *β-actin* as an endogenous control. Data are expressed as mean ± SE for each group (*n* = 4). The statistical analyses were performed using Student’s *t*-test vs. No addition.

In order to see the mechanism of HMGB1- and AGE-induced gene expression of *IL-6* and *CCL2*, *RAGE* gene was knocked down by RNA interference. The expression of *IL-6* and *CCL2* was significantly increased by the addition of HMGB1 and AGE even in the presence of scrambled RNA. In contrast, introduction of small interfering RNA (siRNA) for RAGE (*siRAGE*) clearly inhibited the HMGB1- and AGE-induced increases of mRNAs for *IL-6* and *CCL2* in SW872 human adipocytes (Figure 4; *P* = 0.2638 [No addition vs. HMGB1 in *IL-6*], *P* = 0.0744 [No addition vs. AGE in *IL-6*], *P* = 0.2559 [No addition vs. HMGB1 in *CCL2*], and *P* = 0.5541 [No addition vs. AGE in *CCL2*]).

**Figure 5:**
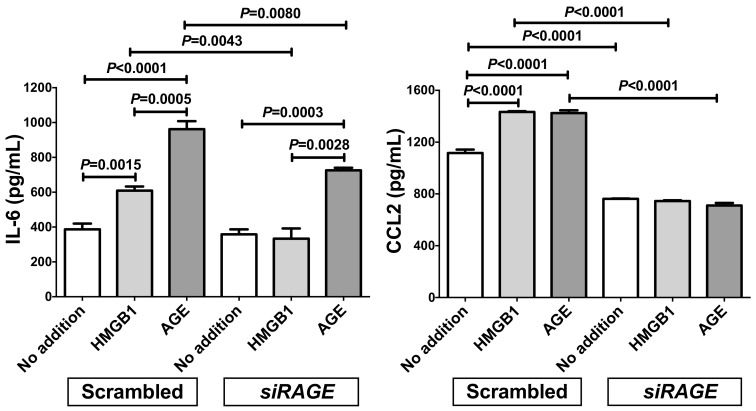
Effect of siRNA against RAGE on the HMGB1- and AGE-induced expression of IL-6 and CCL2. SiRNA for RAGE was transfected into SW872 cells and the cells were incubated with HMGB1 or AGE for 24 h. The levels of IL-6 and CCL2 in the cell culture medium were measured via ELISA. Data are expressed as mean ± SE for each group (*n* = 4). The statistical analyses were performed using Student’s *t*-test vs. No addition.

We also measured the concentrations of IL-6 and CCL2 in the RAGE-knocked-down SW872 cell culture medium via enzyme-linked immunosorbent assay (ELISA). The concentrations of IL-6 and CCL2 were significantly increased in response to HMGB1 and AGE in scrambled RNA-introduced cell culture medium. In contrast, the introduction of siRAGE significantly attenuated the HMGB1- and AGE-induced increases of IL-6 and CCL2 in the medium (Figure 5).

### 2.4. Up-Regulation of IL-6 and CCL2 by Lipopolysaccharide (LPS) in Adipocytes

**Figure 6:**
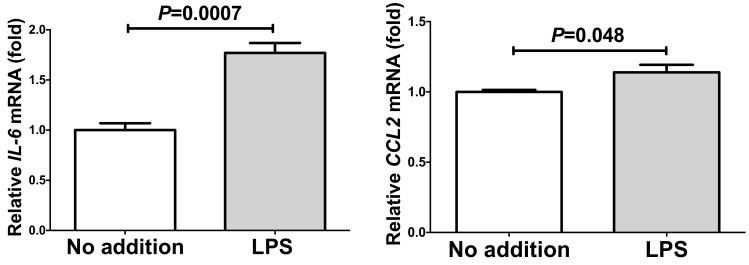
The mRNA levels of IL-6 and CCL2 in SW872 human adipocytes treated with 10 ng/mL lipopolysaccharide (LPS) for 24 h. The levels of the mRNAs were measured via real-time RT-PCR using β-actin as an endogenous control. Data are expressed as mean ± SE for each group (*n* = 4). The statistical analyses were performed using Student’s *t*-test.

Recent reports indicated that PE/HDP is also induced by lipopolysaccharide (LPS) [32] and that RAGE mediates LPS signaling and acts as an LPS receptor [33,34,35,36,37,38]. Thus, we tested whether LPS up-regulate gene expression of *IL-6* and *CCL2* in human SW872 adipocytes. We added 10 ng/mL LPS in SW872 culture medium, incubated for 24 h, and the expression of *IL-6* and *CCL2* was analyzed via real-time RT-PCR. As shown in Figure 6, mRNAs of *IL-6* and *CCL2* were significantly up-regulated by the addition of LPS.

**Figure 7:**
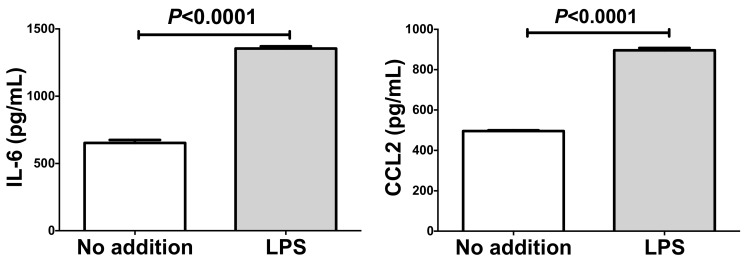
The levels of IL-6 and CCL2 in culture medium of SW872 human adipocytes treated with 10 ng/mL LPS for 24 h. The levels of IL-6 and CCL2 in the cell culture medium were measured via ELISA. Data are expressed as mean ± SE for each group (n = 4). The statistical analyses were performed using Student’s *t*-test.

We next measured IL-6 and CCL2 in the LPS-stimulated SW872 cell culture medium and found that the levels of IL-6 and CCL2 in the LPS-stimulated SW872 culture medium were also elevated significantly (Figure 7).

### 2.5. Down-Regulation of RAGE Attenuated the LPS-Induced IL-6 and CCL2 Increases in Adipocytes

**Figure 8:**
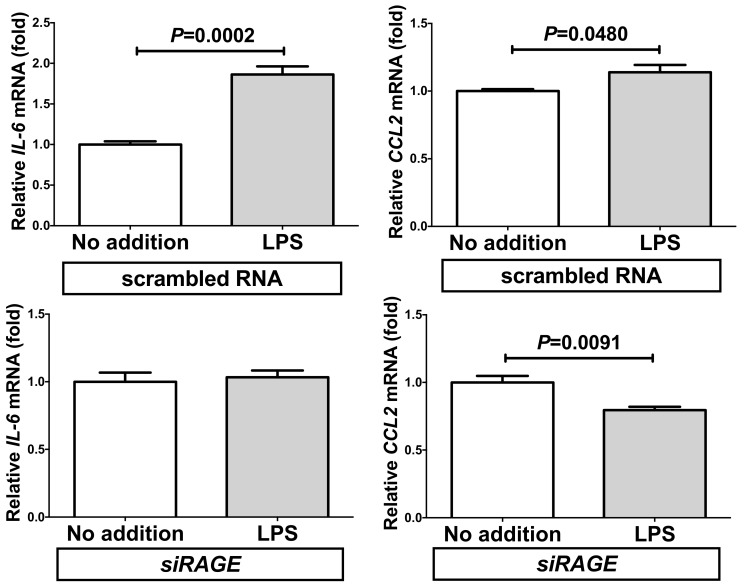
Effects of siRNA against *RAGE* on the LPS-induced gene expression of *IL-6* and *CCL2*. SiRNA for *RAGE* was transfected into SW872 cells and the cells were incubated with 10 ng/mL LPS for 24 h. The levels of *IL-6* and *CCL2* mRNA were measured via real-time RT-PCR using *β-actin* as an endogenous control. Data are expressed as mean ± SE for each group (*n* = 4). The statistical analyses were performed using Student’s *t*-test.

In order to confirm whether the mechanism of LPS-induced *IL-6* and *CCL2* up-regulation is also mediated by RAGE, *RAGE* gene was knocked down by RNA interference. The expression of *IL-6* and *CCL2* was significantly increased by the addition of LPS even in the presence of scrambled RNA. In contrast, introduction of *siRAGE* clearly inhibited the LPS-induced increases of mRNAs for *IL-6* and *CCL2* in SW872 human adipocytes (Figure 8).

**Figure 9:**
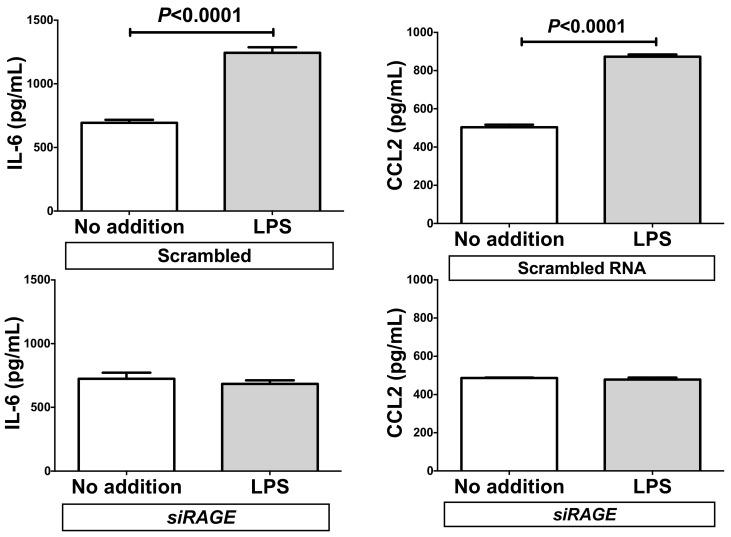
Effect of siRNA against RAGE on the LPS-induced expression of IL-6 and CCL2. SiRNA for RAGE was transfected into SW872 cells and the cells were incubated with LPS for 24 h. The levels of IL-6 and CCL2 in the cell culture medium were measured via ELISA. Data are expressed as mean ± SE for each group (*n* = 4). The statistical analyses were performed using Student’s *t*-test.

We also measured the concentrations of IL-6 and CCL2 in the RAGE-knocked-down SW872 cell culture medium via ELISA. The concentrations of IL-6 and CCL2 were significantly increased in response to the addition of LPS in scrambled RNA-introduced cell culture medium. In contrast, the introduction of siRAGE significantly attenuated the LPS-induced increases of IL-6 and CCL2 in the medium (Figure 9).

## 3. Discussion

Previous studies indicated that body mass index (BMI), anemia, lower education, maternal age, primiparity, multiple pregnancy, PE/HDP in previous pregnancy, gestational diabetes mellitus, preexisting hypertension, preexisting type 2 diabetes mellitus, preexisting urinary tract infection, and a family history of hypertension, type 2 diabetes mellitus, or PE/HDP are potential risk factors for PE/HDP [39,40]. Of the risk factors, obesity is a major risk factor and is associated with an increased risk for obstetrical complications such as gestational diabetes mellitus, PE/HDP, pre-term delivery, and Cesarean section [41,42,43,44,45], and increased neonatal morbidity and mortality [42,46,47]. Maternal obesity has been associated with low-grade metabolic inflammation due to increased release of adipokines, which are believed to contribute to maternal glucose intolerance and insulin resistance and cardiovascular and neuroendocrine modulation associated with increased maternal BMI [48]. Increased cytokine and decreased adiponectin release from adipose tissue have been linked to the meta-inflammatory state of obesity [49,50].

In this study, we measured the mRNA levels for adipokines (*LEP*, *ADIP*, and *RETN*) in human primary adipocytes and found that they were not up-regulated in response to the addition of sera from PE/HDP patients. We also measured mRNA levels of *TNFα*, *IL-6*, and *CCL2,* which have been reported to play important roles in pathogenesis or development of PE/HDP, and found that the expression of *IL-6* and *CCL2* was elevated in response to the addition of PE/HDP sera. In addition, the mRNA levels of RAGE system members (*HMGB1*, *S100B*, and *RAGE*) were significantly elevated by the addition of PE/HDP patient sera, suggesting possible involvement of the RAGE system in the up-regulation of *IL-6* and *CCL2* in adipocytes. In order to verify this possibility, we tested whether RAGE ligands up-regulate expression of *IL-6* and *CCL2* using human SW872 adipocytes and mouse 3T3-L1 cells and found that AGE and HMGB1 but not S100B significantly up-regulated gene expression of *IL-6* and *CCL2* in SW872 cells. In contrast to SW872 cells, AGE and HMGB1 up-regulated the gene expression of *IL-6* in differentiated 3T3-L1 cells but not in undifferentiated cells, and the addition of AGE, but neither HMGB1 nor S100B, up-regulated *Ccl2* expression in undifferentiated 3T3-L1 cells but any of them up-regulated *Ccl2* in differentiated cells. These results indicate that RAGE ligands, especially AGE and HMGB1, stimulate adipocytes to induce gene expression of *IL-6* and *CCL2*.

IL-6 is a key player in tissue inflammation and insulin resistance, and was observed in higher serum concentrations in PE/HDP patients [51]. CCL2, also referred as monocyte chemoattractant protein-1, is a key regulator of monocyte infiltration of adipose tissue, and it plays a central role in the development and maintenance of chronic adipose tissue inflammation and insulin resistance [23,52,53]. Therefore, IL-6 and CCL2 could be key players produced from adipocytes to induce tissue damages in PE/HDP patients.

Exposure of the amino acid residues of proteins to reducing sugars, such as glucose, results in non-enzymatic glycation, which forms reversible Schiff bases and subsequently Amadori compounds. A series of further complex molecular rearrangements including dehydration, condensation, and crosslinking, yield irreversible and heterogeneous derivatives termed AGE. AGEs are chemically heterogeneous groups of compounds. Apart from endogenously formed AGEs, exogenous AGEs from foods are absorbed in the gastrointestinal tract and reportedly constitute ~10% of total AGE in the body. In animal studies, the restriction of dietary AGE intake significantly improved insulin sensitivity and extended lifespan.

HMGB1 is a nuclear protein that stabilizes nucleosome formation and facilitates transcription. HMGB1 is a strong inflammatory trigger from necrotic cells as a result of passive leakage, and can be actively secreted by activated monocytes, macrophages, dendritic cells, natural killer cells, and endothelial cells, though there is no canonical signal sequence in the HMGB1 protein. It is well-known that the levels of AGE in serum such as hemoglobin A1c (HbA1c) are increased in diabetes (hyperglycemia) patients and that diabetes is a typical risk factor for PE/HDP. Elevated HMGB1 was observed in pregnant women with other pro-inflammatory conditions as obesity and pre-term labor. It is well established that labor is associated with a pro-inflammatory systemic response. Extracellular HMGB1 exerts its cytokine-like activity by binding to RAGE receptor. In fact, the serum HMGB1 levels were significantly increased in the PE/HDP patients (329.2 ± 93.18 ng/mL) than those in control patients (35.45 ± 25.11 ng/mL) (*P* = 0.0473). In the management of pregnant women, monitoring of blood glucose and HbA1c are very common but HMGB1 levels in serum are rarely monitored. Although the numbers of PE/HDP patients in our study were relatively small, the increased tendency of serum HMGB1 in PE/HDP patients suggests that the serum HMGB1 measuring could be a new marker for screening of PE/HDP risk.

As AGE and HMGB1 are ligands for RAGE, it is quite possible that AGE- and HMGB1-induced up-regulation of *IL-6* and *CCL2* is mediated via RAGE. In fact, the introduction of *siRAGE* abolished the AGE- and HMGB1-mediated increases of gene expression of *IL-6* and *CCL2* in adipocytes (Figure 4 and Figure 5), indicating involvement of AGE and/or HMGB1/RAGE system in the up-regulation of IL-6 and CCL2 in adipocytes. Among major RAGE ligands, we tested S100B, in addition to AGE and HMGB1, but S100B failed to increase gene expression of *IL-6* and *CCL2*. As most but not all the ligands for RAGE up-regulate (pro)inflammatory mediators, such as IL-6 and CCL2, some other RAGE ligands such as macrophage-1 antigen/cluster of differentiation molecule 11b [54], amyloid β peptide [55], β-sheet fibrils [56], advanced oxidation protein products [57], complement C3a [58], LPS [33], and phosphatidylserine on the surface of apoptotic cells [59] might increase the expression of IL-6 and CCL2, leading to PE/HDP in pregnant women. In fact, recent reports showed that PE/HDP was also induced by LPS [32], and that RAGE mediated LPS signaling and acted as an LPS receptor [33,34,35,36,37,38]. Thus, we tested whether LPS up-regulate gene expression of IL-6 and CCL2 in human SW872 adipocytes, and found that LPS significantly up-regulated the expression of IL-6 and CCL2 in SW872 cells via RAGE (Figure 6, Figure 7, Figure 8 and Figure 9).

Some soluble products of RAGE such as soluble RAGE (sRAGE) and endogenous secretory RAGE (esRAGE) are generated from *RAGE* gene and modulate the RAGE signaling [60,61]. It was previously reported that the levels of sRAGE were reduced in PE/HDP patient serum and that serum esRAGE and the esRAGE/sRAGE ratio were elevated in PE/HDP patient serum [62]. It was also reported that pregnancy induced a significant increase in RAGE protein levels in both myometrium and omental vasculature and that blood vessels from women with preeclampsia had intense staining for RAGE in both vessel beds [63]. In the present study, we showed the up-regulation of RAGE in adipocytes by PE/HDP sera (Figure 1) but did not see sRAGE and esRAGE. Reduction of sRAGE and elevation of esRAGE/sRAGE ratio could be a potential marker for screening of PE/HDP risk.

Nuclear factor κ-light-chain-enhncer of activated B cells (NF-κB) is a key transcription factor for the expression of IL-6 and CCL2 [64,65]. RAGE ligands usually activate NF-κB [66]. The RAGE-NF-κB-IL-6/CCL2 pathway might function in adipocytes stimulated by RAGE ligands (AGE, HMGB1, and LPS), resulting in the development of inflammation that may lead to PE/HDP in pregnant women.

## 4. Materials and Methods

### 4.1. Patient Samples

The study was approved by the Local Ethics Committee at Nara Medical University (Kashihara, Japan; approval number 873, 24 July 2014), and all participants provided written informed consent. We included PE/HDP patients with a pregnancy and disease-free pregnant women with pregnancy were the control (Table 1). The participants’ BMI values before pregnancy were less than 25 kg/m^2^ with gestational age-matched normal pregnant women at 27 weeks’ gestation or later. All subjects were Eastern Asian origin, and none of the subjects were taking any medication or showed evidence of any metabolic diseases or other complications besides PE/HDP. PE/HDP was defined as new onset and diagnosed based on two consecutive measurements of diastolic and systolic blood pressure, diastolic blood pressure greater than or equal to 90 mmHg, or systolic blood pressure was greater than or equal to 140 mmHg, with urine protein over 300 mg/day, occurring diagnosed after 20 weeks of gestation [67]. All subjects (4 patients and 4 controls) provided serum samples for analysis and did not have gestational diabetes mellitus, thyroid malfunction, or other complications. All venous blood samples were obtained after an overnight fast at routine medical examination. The sera were separated immediately and stored at −80 °C for 3 years at the longest and 6 months at the shortest. HMGB1 concentrations of the sera were measured using Human HMGB1 ELISA kit (Arigo Biolaboratories Corp., Hsinchu, Taiwan).

### 4.2. Cell Culture and Treatment

Human primary visceral preadipocytes were purchased from ZenBio, Inc. (Research Triangle Park, NC, USA). The cells were differentiated to adipocytes according to the supplier’s protocol, and their differentiation to mature adipocyte was confirmed by Oil Red O staining. The primary adipocytes were incubated with 10% individual PE/HDP patient serum (#1~#4) and control serum (#1~#4) for 24 h. Human SW872 adipocytes were purchased from American Type Culture Collection (ATCC, Manassas, VA), and cultured at 37 °C with 5% CO_2_ in DMEM medium (Wako Pure Chemical Industries, Ltd., Osaka, Japan) supplemented with 10% fetal calf serum (FCS), 100 units/mL penicillin G (Wako) and 100 μg/mL streptomycin (Wako) as described [68]. Mouse 3T3-L1 preadipocytes were purchased from Japanese Collection of Research Bioresources (JCRB) Cell Bank (Ibaraki, Japan), and cell culture and differentiation of 3T3-Ll preadipocytes were performed as described by Ntambi et al. [69,70]. Briefly, confluent 3T3-Ll pre-adipocytes monolayers were incubated for 72 h in DMEM medium containing 10% FCS, 0.5 mM methylisobutylxanthine (IBMX; Wako), 1 µM dexamethasone (Wako), and 10 µg/mL insulin (Wako). After 72 h the cells were washed free of IBMX and dexamethasone and maintained in DMEM medium containing 10% FCS and 10 µg/mL insulin for 72 h. Adipocyte morphology was monitored by the appearance of cytoplasmic triacylglycerol droplets, which is closely correlated with the acquisition of the adipocyte phenotype. For the stimulation experiments, SW872 and 3T3-L1 cells (undifferentiated preadipocytes and differentiated adipocytes) were treated with 150 µg/mL (for SW872) or 300 µg/mL (for 3T3-L1) AGE-bovine serum albumin (BSA) (Calbiochem^®^, Merck KGaA, Darmstadt, Germany), 1 µg/mL HMGB1 (Bio-Techne, Minneapolis, MN) or 100 ng/mL S100B (Medical & Biological Laboratories Co., Ltd., Nagoya, Japan). SW872 cells were also treated with 10 ng/mL *E. coli* LPS (Wako) for 24 h as described [33].

### 4.3. Real-Time Reverse Transcriptase-Polymerase Chain Reaction (RT-PCR)

Total RNA was isolated using a RNeasy Protect Cell Mini Kit (Qiagen, Hilden, Germany) from primary cultured human visceral adipocytes, SW872, and 3T3-L1 adipocytes/preadipocytes, and cDNA was synthesized from total RNA as template using a High Capacity cDNA Reverse Transcription kit (Applied Biosystems, Foster City, CA) as described [68,70,71,72,73,74,75,76,77,78,79,80,81,82]. Real-time polymerase chain reaction (PCR) was performed using SYBR^®^ Fast qPCR kit (KAPA Biosystems, Boston, MA) and a Thermal Cycler Dice Real Time System (Takara Bio Inc., Kusatsu, Japan). All the PCR primers were synthesized by Nihon Gene Research Laboratories, Inc. (NGRL; Sendai, Japan), and the primer sequences for each primer set are described in Table 2. PCR was performed with an initial step of 3 min at 95 °C followed by 40 cycles of 3 s at 95 °C and 20 s at 60 °C for human *β-actin*, mouse *rat insulinoma gene (Rig)/ribosomal protein S15* (*RpS15*), mouse *IL-6*, human and mouse *CCL2*, human *LEP*, human *ADIP*, human *RETN*, human *S100B,* human *HMGB1,* and human *RAGE*, and with an initial step of 3 min at 95 °C followed by 40 cycles of 3 s at 95 °C and 20 s at 62 °C for human *IL-6* and human *TNFα*. The mRNA expression levels were normalized to the mRNA level of *Rig/RpS15* in mouse samples or *β-actin* in human samples [68,70,71,72,73,74,75,76,77,78,79,80,81,82,83,84,85].

### 4.4. Measurement of IL-6 and CCL2 Concentrations in Culture Medium via ELISA

Cells were stimulated with HMGB1 (1 µg/mL), AGE (150 and 300 µg/mL), S100B (100 ng/mL), and LPS (10 ng/mL) for 24 h, culture medium was collected, and the concentrations of IL-6 and CCL2 were measured by using a Human IL-6 ELISA kit (RayBiotech, Norcross, GA, USA) for IL-6 and a Quantikine^®^ ELISA Human CCL2/MCP-1 Immunoassay kit (R&D Systems, Inc., Minneapolis, MN, USA) for CCL2, according to the instructions of suppliers.

### 4.5. RNA Interference (RNAi)

Small interfering RNA (siRNA) directed against human *RAGE* was synthesized by NGRL. The sense sequence of siRNA for human *RAGE* was 5′-AUCUACAAUUUCUGGCUUCtt-3′ (corresponding to 466-484 of NM_001136) as described [76,78]. The Silencer^®^ Select human scrambled siRNA was purchased from Ambion^®^ (Waltham, MA, USA) and used as a control. Transfection of siRNAs to SW872 cells was carried out using Lipofectamine^®^ RNAiMAX Reagent (Life Technologies, Waltham, MA, USA) as described [68,72,74,75,76,77,78,79,80]. Cells were transfected with 5 pmol siRNA per 24-well culture dish (4.0 × 10^5^ cells/mL in 24-well plates).

### 4.6. Data Analysis

Results are expressed as mean ± SE. The data obtained were checked against Shapiro-Wilk normality test, which found that all the *P* values were larger than 0.05, and the statistical significance was determined by Student’s *t*-test using GraphPad Prism ver. 6.0 for Mac OSX software (GraphPad Software, La Jolla, CA, USA).

## Figures and Tables

**Table 1:** Clinical characteristics of patients/controls involved in the study.

Patients/Controls	Age (Years)	BMI	Gestational Age at Blood Sampling (Week)	Parity
PE/HDP #1	33	23.2	30	0
PE/HDP #2	27	21.9	29	1
PE/HDP #3	28	21.3	28	0
PE/HDP #4	29	23.4	27	0
Control #1	30	22.4	28	0
Control #2	29	24.6	28	0
Control #3	26	23.8	27	0
Control #4	33	22.4	28	2

**Table 2:** Primers used for real-time reverse transcriptase-polymerase chain reaction (RT-PCR).

Target mRNA	Primer Sequence (Position)
Human *IL-6* (NM_000600)	5′-GGTACATCCTCGACGGCATC-3′ (289–308) 5′- GCCTCTTTGCTGCTTTCACAC-3′ (347–367)
Human *CCL2* (NM_002982)	5′-GTCTCTGCCGCCCTTCTGT-3′ (80–98) 5′-TTGCATCTGGCTGAGCGAG-3′ (137–155)
Human *TNFα* (NM_000594)	5′-CTTCTCCTTCCTGATCGTGG-3′ (280–299) 5′-TCTCAGCTCCACGCCATT-3′ (518–535)
Human *LEP* (NM_000230)	5′-GGCTTTGGCCCTATCTTTTC-3′ (89–108) 5′-GGATAAGGTCAGGATGGGGT-3′ (257–276)
Human *ADIP* (NM_001177800)	5′-CATGACCAGGAAACCACGACT-3′ (181–201) 5′-TGAATGCTGAGCGGTAT-3′ (465–481)
Human *RETN* (NM_020415)	5′-TCCTCCTCCTCCCTGTCCTGG-3′ (63–83) 5′-CAGTGACATGTGGTCTGGGCG-3′ (298-318)
Human *S100B* (NM_006272)	5′-AGGGAGGGAGACAAGCACAA-3′ (172–191) 5′-ACTCGTGGCAGGCAGTAGTA-3′ (293–312)
Human *HMGB1* (NM_001313893)	5′-ATATGGCAAAAGCGGACAAG-3′ (1126–1145) 5′-AGGCCAGGATGTTCTCCTTT-3′ (1281–1300)
Human *RAGE* (NM_001136)	5′-TGGAACCGTAACCCTGACCT-3′ (856–875) 5′-CGATGATGCTGATGCTGACA-3′ (1045–1064)
Human *β-actin* (NM_001101)	5′-GCGAGAAGATGACCCAGA-3’ (420–437) 5´-CAGAGGCGTACAGGGATA-3´ (492–509
Mouse *IL-6* (NM_031168)	5′-GTATGAACAACGATGATGCACTTG-3′ (305–328) 5′-ATGGTACTCCAGAAGACCAGAGGA-3′ (418–441)
Mouse *Ccl2* (NM_011333)	5′-CCACTCACCTGCTGCTACTCAT-3′ (176–197) 5′-TGGTGATCCTCTTGTAGCTCTCC-3′ (229–251)
Mouse *Rig/RpS15* (NM_009091)	5′-ACGGCAAGACCTTCAACCAG-3′ (323–342) 5′-ATGGAGAACTCGCCCAGGTAG-3′ (372–392)

## Abbreviations

ADIP	Adiponectin
AGE	Advanced glycation endproduct(s)
BMI	Body mass index
CCL2	C-C motif chemokine ligand 2
ELISA	Enzyme-linked immunosorbent assay
esRAGE	Endogenous secretory RAGE
FCS	Fetal calf serum
HbA1c	Hemoglobin A1c
HMGB1	High mobility group box 1
IBMX	Methylisobutylxanthine
IL-6	Interleukin-6
LEP	Leptin
LPS	Lipopolysaccharide
NF-κB	Nuclear factor κ-light-chain-enhancer of activated B cells
PCR	Polymerase chain reaction
PE/HDP	Preeclampsia/hypertensive disorders of pregnancy
RAGE	Receptor for advanced glycation endproduct(s)
RETN	Resistin
Rig/RpS15	Rat insulinoma gene/Ribosomal protein S15
RT-PCR	Reverse transcriptase-PCR
S100B	S100 Ca^2+^-binding protein B
siRNA	Small interfering RNA
sRAGE	Soluble RAGE
TNFα	Tumor necrosis factor α
